# Strategies for developing long-lasting therapeutic nucleic acid aptamer targeting circulating protein: The present and the future

**DOI:** 10.3389/fcell.2022.1048148

**Published:** 2022-11-01

**Authors:** Yihao Zhang, Huarui Zhang, Daniel Wing Ho Chan, Yuan Ma, Aiping Lu, Sifan Yu, Baoting Zhang, Ge Zhang

**Affiliations:** ^1^ Law Sau Fai Institute for Advancing Translational Medicine in Bone and Joint Diseases, School of Chinese Medicine, Hong Kong Baptist University, Kowloon, Hong Kong SAR, China; ^2^ School of Chinese Medicine, Faculty of Medicine, The Chinese University of Hong Kong, Hong Kong, China; ^3^ Institute of Integrated Bioinfomedicine and Translational Science, School of Chinese Medicine, Hong Kong Baptist University, Kowloon, Hong Kong SAR, China; ^4^ Institute of Precision Medicine and Innovative Drug Discovery, HKBU Institute for Research and Continuing Education, Shenzhen, China

**Keywords:** aptamer, half-life, long-lasting modification, PEGylation, low molecular weight coupling agent

## Abstract

Aptamers are short, single-stranded DNA or RNA oligonucleotide sequences that can bind specific targets. The molecular weight of aptamers (<20 kDa) is lower than the renal filtration threshold (30∼50 kDa), resulting in very short half-lives *in vivo*, which limit their druggability. The development of long-lasting modification approaches for aptamers can help address the druggability bottleneck of aptamers. This review summarized two distinct kinds of long-lasting modification approaches for aptamers, including macromolecular modification and low-molecular-weight modification. Though it is a current approach to extend the half-life of aptamers, the macromolecular modification approach could limit the space for the dosage increases, thus causing potential compliance concerns due to large molecular weight. As for the other modification approach, the low-molecular-weight modification approach, which uses low molecular weight coupling agents (LMWCAs) to modify aptamers, could greatly increase the proportion of aptamer moiety. However, some LMWCAs could bind to other proteins, causing a decrease in the drug amounts in blood circulation. Given these issues, the outlook for the next generation of long-lasting modification approaches was proposed at the end, including improving the administration method to increase dosage for aptamer drugs modified by macromolecule and developing Artificial intelligence (AI)-based strategies for optimization of LMWCAs.

## 1 Introduction

Aptamers are short, single-stranded DNA or RNA (ssDNA or ssRNA) oligonucleotide sequences that can bind specific targets such as peptides, proteins, small molecules, and even live cells, usually consisting of 25–80 nucleotides. Systematic evolution of ligands by exponential enrichment (SELEX) was first developed in 1990 by Tuerk and Gold (1990) and Ellington and Szostak (1990), separately. It is used to screen functional nucleic acids *in vitro*. Nucleic acid ligands generated by using SELEX are termed aptamers. They have diverse three-dimensional structures and the ability to play a role similar to monoclonal antibodies while reducing immunogenicity and production costs compared to monoclonal antibodies. The differences between aptamers and monoclonal antibodies are summarized in Table 1 (Marangoni et al., 2015; Oelkrug et al., 2015; Dunn et al., 2017). DNA synthetic methods have advanced in recent years, thus generating a large population of degenerate oligodeoxynucleotides (Rothlisberger and Hollenstein, 2018). Besides, due to the advances of PCR, the small numbers of DNA molecules selected from DNA library can be easily magnified into amounts that researchers can readily manipulate. Thanks to the two advances mentioned above, researchers got the ability to partition oligonucleotides based on their binding or catalytic activities.

**TABLE 1:** Differences between aptamers and monoclonal antibodies.

	Aptamer	Monoclonal antibody
Size	∼12–30 kDa	∼150–170 kDa
Manufacturing	Chemical synthesis	Cell culture
Stability	Chemically stable in a wide range of temperatures; reversibility of folding	Susceptible to high temperatures and pH changes; generally non-reversible folding
Immunogenicity	Non-immunogenic	Unwanted immunogenicity
Modifiability	Easily modify without affinity loss	Conjugated with signaling or binding molecule

**FIGURE 1:**
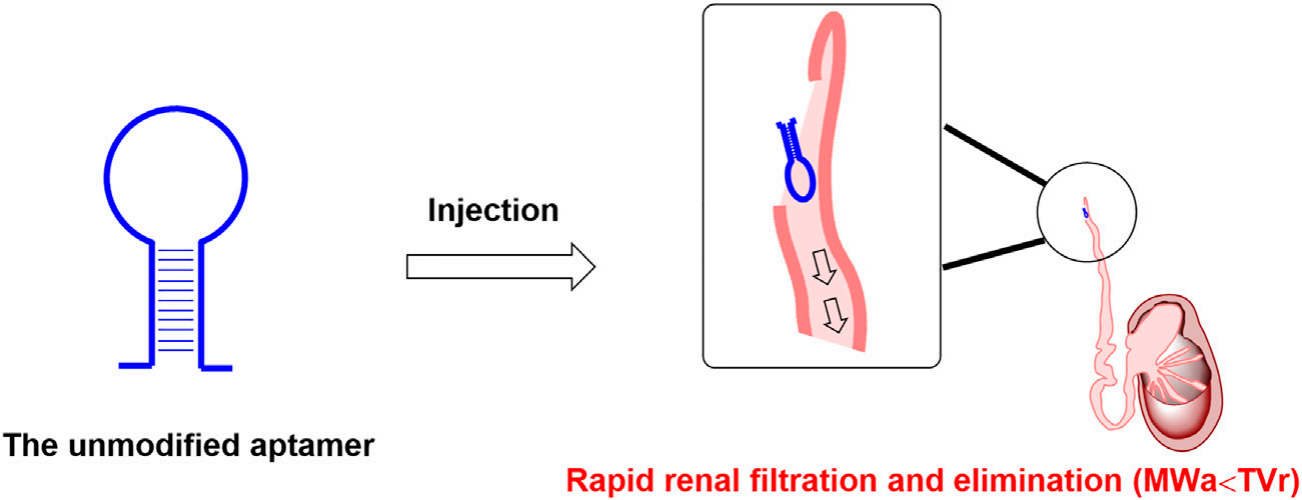
Rapid renal filtration and elimination of aptamer due to short half-life. Note: MW_a_ is short for the molecular weight of the above mentioned unmodified aptamer; TV_r_ is short for the cut-off threshold value of the renal filtration.

In recent years, some aptamers are internalized upon binding to specific cellular receptors, which can be used as targeted agents for small interfering RNAs, microRNAs, conventional drugs, etc. (Tan et al., 2013; Nozari and Berezovski, 2017; Wang et al., 2018), which drove scientists to develop a large amount of diverse applied paradigms in therapeutic research. Owing to the chemical synthesis properties, aptamers are usually easy to be coupled with specific carriers such as liposomes to form a targeted drug delivery system, thus enabling small molecules, nucleic acids, and peptides to be modified and engineered to targeted delivery agents (Liang et al., 2017). However, unmodified nucleic acid aptamers are susceptible to nuclease-mediated cleavage and rapid excretion through renal filtration (Figure 1), which makes nucleic acid aptamers rapidly eliminated and hold a very short biological half-life *in vivo*. These pose significant problems, seriously hampering the nucleic acid aptamer’s clinical transformation. In order to increase the circulation half-life, a number of attempts were reported to circumvent this challenge, with the main focus on the following two methods. Firstly, various chemical modifications can be applied to nucleic acid aptamer’s backbone to avoid enzymatic degradation. Similar to monoclonal antibodies, after some essential chemical modifications to improve the resistance to the nuclease degradation and prolong the biological half-life, inhibitory aptamers that can disrupt the function of pathological target proteins can be used directly as therapeutic agents (Wei and Cohen, 2019). Secondly, several low molecular weight coupling agents (LMWCAs) can be chemically conjugated to the nucleic acid aptamer structure to achieve the long-lasting demand. These LMWCAs could bind some long-half-life proteins (like albumin) and form a protein/LMWCA-aptamer complex. Therefore, the circulation half-life of the conjugated aptamers could be prolonged. In this review, we summarized two distinct kinds of long-lasting modification approaches for aptamers, one is macromolecular modification, and the other is low-molecular-weight modification. In general, macromolecular modification like PEGylation is a useful approach to extending the circulation half-life of aptamers *in vivo*. However, this approach could limit the space for dosage increases, thus causing potential compliance concerns due to large molecular weight. Although the low-molecular-weight modification approach could greatly increase the proportion of aptamer moiety, some LMWCAs could not only bind albumin but also bind other plasma proteins, decreasing the drug amounts in blood circulation. Considering the issues mentioned above, the perspective of the next generation of long-lasting modification approaches was proposed at the end of the manuscript, including improving the administration method to increase dosage for aptamer drugs modified by macromolecule and developing artificial intelligence (AI)-based strategies for optimization of LMWCAs.

## 2 Current approaches for developing long-lasting nucleic acid aptamers

Two processes mainly limit the biological half-life of nucleic acid aptamers. One is the specific degradation process of nucleases, which can be overcome by chemical modifications like the 2′-fluoro (2′-F) modification on the sugar ring (Ni et al., 2017). The other is the renal clearance process. The average diameter of aptamers with a mass of 6–30 kDa is less than 5 nm (Guo, 2010). Non-formulated small aptamers or aptamers using stabilizing backbone modifications are subject to rapid excretion through renal filtration when administered into the blood. Thus, the modification with bulky moiety helps to avoid renal filtration and extends circulation half-life. Most recently, several approaches have been reported to avoid the renal clearance process by attaching a bulky moiety, such as cholesterol (Lee et al., 2015), polyethylene glycol (PEG) (Burmeister et al., 2005), proteins (Heo et al., 2016), liposomes (Willis et al., 1998), organic or inorganic nanomaterials (Zhou et al., 2009), at the 5′-end of aptamers. Among these approaches, PEGylation (Abbina and Parambath, 2018) is one of the most classical strategies. PEG is a synthetic homopolymer for medicinal injection approved by U.S. Food and Drug Administration. Drug molecules’ half-life can be increased through conjugation with PEG. Other strategies are also used to optimize the metabolic stability and pharmacokinetic properties of therapeutic nucleic acid aptamers, such as their conjugation with lipid nanoparticles delivery system (Samaridou et al., 2020) or to bioactive natural protein (Hoogenboezem and Duvall, 2018). Alternatively, long-half-life proteins and LMWCAs are also reported to extend the circulation half-life of aptamers.

### 2.1 Nucleic acid aptamer modified by macromolecules

#### 2.1.1 PEG

**FIGURE 2:**
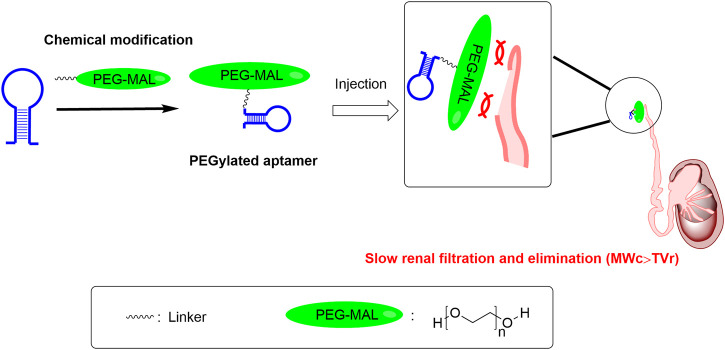
The PEGylation approach can help address druggability bottleneck of aptamers with short half-life. Note: MW_c_ is short for the molecular weight of the above mentioned PEG-conjugated aptamer; TV_r_ is short for the cut-off threshold value of the renal filtration. PEG-MAL is short for the polyethylene glycol maleimide.

PEGs are high-molecular-weight synthetic polymers, linear or branched, containing an elevated number of oxygen atoms of ether type (polyether) (Turecek et al., 2016). The molecular weight of PEG ranges from a few hundred to tens of thousands, and its physicochemical properties will correspondingly have different attributes. PEG with a molecular weight lower than 700 is liquid at room temperature, while PEG with a molecular weight higher than 1,000 is mostly in the form of a solid (Raees et al., 2018). PEG is approved by U.S. Food and Drug Administration for use in pharmaceuticals. It has low toxicity and low immunogenicity. PEG with a molecular weight lower than 30kDa is usually eliminated from the body by the kidney, while PEG with a molecular weight higher than 20kDa is usually excreted in the feces (Harris and Chess, 2003). The attachment of PEG to macromolecules like proteins, peptides, and nucleic acids is called PEGylation. After PEGylation, PEG forms a hydrophilic shield on the surface of their drug molecules, which can reduce immune recognition *in vivo* and their degradation by enzymes. Meanwhile, PEGylation increases the size of drug molecules, thereby decreasing their renal clearance, as the renal clearance mostly depends on the size. Drugs with PEGylation usually have better efficacy because of the extended biological half-life. PEGylation is also used in extending therapeutic aptamers’ biological half-life, shown in several examples below (Figure 2).

PEG molecules with different molecular weights differ in their effect on extending biological half-life. For instance, the first generation of the von Willebrand factor (VWF) aptamer ARC1779 was a PEGylated aptamer that was generally safe and effective. A PEG moiety with a molecular weight of 20 kDa was conjugated to ARC1779 aptamer to reduce renal clearance (Diener et al., 2009). The half-life of ARC1779 *in vivo* was approximately 2 hours (Gilbert et al., 2007), which greatly limited its druggability. To further extend the half-life of the VWF aptamer, the second generation of the VWF aptamer ARC15105 with higher PEG moieties (40kDa) was designed, which possessed a longer half-life *in vivo* (66 h) and showed comparable inhibition of VWF activity (Siller-Matula et al., 2012).

Pegaptanib is the first pegylated RNA aptamer approved by U.S. Food and Drug Administration for its use in age-related macular degeneration. Its trade name is Macugen, developed in collaboration between Eyetech and Pfizer in 2004. Pegaptanib binds specifically to the extracellular target VEGF165 and inhibits the binding of VEGF165 to VEGF receptors, thereby inhibiting neoangiogenesis. Pegaptanib is modified by 40 kDa PEG (Fishburn, 2008). In a safety-pharmacokinetic trial (Macugen et al., 2007), the result shows that it has a half-life of approximately 10 days. In a phase II, randomized, multicenter, controlled, double-blind clinical trial (Gragoudas et al., 2004), 70% of patients applicated 0.3mg pegaptanib for 1 year maintained stable visual acuity. Throughout the clinical trial, the mean visual acuity of the patients given pegaptanib was always better than those of the blank group. In other words, patients given pegaptanib showed a significant treatment effect compared with the controls. The clinical trial also shows that pegaptanib has some adverse effects, such as endophthalmitis, traumatic injury to the lens, and retinal detachment.

Olaptesed pegol (NOX-A12) is a PEGylated RNA Spiegelmer (L-ribonucleic acid aptamer) that specifically binds to the chemokine CXCL12 and inhibits the binding of CXCL12 to CXCR4 and CXCR7, thereby inhibiting the angiogenesis and metastasis from assisting the cancer treatment. NOX-A12 is modified by 40 kDa branched PEG (Vater et al., 2013). A phase I/II clinical trial (Steurer et al., 2019) was carried out to study the safety, pharmacokinetics properties, pharmacodynamics properties, and therapeutic effect of the combined use of NOX-A12, bendamustine, and rituximab. The pharmacokinetic result shows that NOX-A12 has a half-life of 53.2 h in patients given 4 mg/kg NOX-A12. Lexapeptid pegol (NOX-H94) is another PEGylated RNA Spiegelmer, which can bind specifically to human hepcidin and inactivates hepcidin’s biological activity, thereby upregulating serum iron concentration and transferrin saturation. NOX-H94 is also modified by 40 kDa branched PEG (Schwoebel et al., 2013). A placebo-controlled clinical trial (Boyce et al., 2016) was carried out to study the safety, pharmacokinetic properties, and pharmacodynamic properties. The result shows that PEGylated NOX-H94 can increase serum iron concentration and transferrin saturation dose-dependently, and the half-life of this PEGylated aptamer ranges from 14.1 h to 26.1 h as the i.v. administration dose increase from 0.3 mg/kg to 4.8 mg/kg.

Recently, our research group used PEG with a molecular weight of 40,000 to conjugate the sclerostin aptamer aptscl56 (Yu et al., 2022b; Wang et al., 2022). The conjugated aptamer was named Apc001PE, which could specifically target the loop3 of sclerostin, thus promoting the bone formation and preserving the sclerostin’s cardiovascular protective effect. A mice experiment was carried out to investigate the pharmacokinetic properties of unmodified aptscl56 and Apc001PE. The result shows that unmodified aptscl56 had a short circulation half-life of 0.8 h, while the Apc001PE possessed a longer circulation half-life of about 58 h (Wang et al., 2022).

PEGylation can also be applied to liposomes (Hatakeyama et al., 2013). However, the PEGylated liposomes will no longer process the extended biological half-life when the liposomes are injected repeatedly (Abu Lila and Ishida, 2019) due to accelerated blood clearance phenomena (Abu Lila et al., 2013). In addition to the previously mentioned methods that have been applied to extend nucleic acid aptamers’ half-life, alternatives to PEGylation that are not currently applied to nucleic acid aptamers but have applications in proteins and other nucleic acids are likely to be applied to extend nucleic acid aptamers’ half-life exploiting similar mechanism, such as PAS (Proline, Alanine, and Serine Sequences) (Schlapschy et al., 2013), PLGA (Poly (lactic-co-glycolic acid)) (Kapoor et al., 2015), etc.

#### 2.1.2 Alternatives to PEG

PAS are synthetic, uncharged, high-molecular-weights polypeptides composed of the repetitive sequence Pro, Ala, and/or Ser. It is highly soluble in water and shows high stability in plasma and no immunogenicity. In the design of PAS sequences (Anand and Vallooran, 2018), residue repeats should be avoided to prevent the formation of secondary structures. Without secondary structures, the PAS sequences show a disordered random coil conformation, which imparts PAS biophysical properties similar to PEG. Due to the physicochemical properties of PAS, drug molecules conjugated with PAS process an increased hydrodynamic volume, which can slow the speed of renal clearance. The anchoring of drug molecules to PAS is called PASylation (Binder and Skerra, 2017). PASylation has similar advantages as PEGylation, such as increasing stability, prolonging the plasma half-life by enlarging the molecular size, avoiding the recognition of enzymes, etc. However, unlike PEG, PAS is quickly degraded by the intracellular proteases when taken into cells, which can avoid the deposition of PAS in vacuoles.

PASylation is superior to PEGylation in many aspects, but it has not been directly applied to therapeutic nucleic acids. According to a recent report (Thomas et al., 2022), liposomes with PEGylation can inhibit the interaction between liposomes and cells. Meanwhile, the PEGylation also inhibits the recognition of the target and the internalization of the nanocarrier (Hatakeyama et al., 2013). PASylation was used as an appropriate alternative strategy to deal with unintended inhibition. In addition, PAS sequences can also be designed into a linker or a spacer of antibody-drug conjugate or bifunctional drug molecules (Lerchner et al., 2016).

PLGA are synthetic polymers with low toxicity, degradability, and biocompatibility. PLGA is made from the polymerization of two monomers, lactic acid (LA) and glycolic acid (GA) (Kapoor et al., 2015). PLGA is approved by U.S. Food and Drug Administration for therapeutic applications. The biological half-life of PLGA ranges from days to years as the molecular weight changes. The degradation products of PLGA, LA, and GA, can be degraded by human metabolism and excreted as carbon dioxide and water. An ideal nanocarrier must have several characteristics, such as safety, biodegradability, biocompatibility, and high loading capacity. Due to its advantages, PLGAs are widely used in drug delivery systems (Chereddy et al., 2016).

It is reported (Tahara et al., 2008) that a PLGA nanosphere drug delivery system can deliver all kinds of nucleic acids by using the emulsion solvent diffusion (ESD) method. Nucleic acids are treated with cationic compounds to form ionic nucleic acid complexes so that nucleic acids can be dissolved into organic solvents, thereby completing the ESD reaction to construct a drug delivery system under mild conditions. The ESD method avoids using ultrasonication in traditional methods, thereby preventing nucleic acids from being destroyed during the reaction. As a result, the ESD method applies to various nucleic acids. For example, Mosafer et al. synthesized an AS1411 aptamer-PLGA nanoparticle targeting glioma cells using a multiple ESD method (Mosafer et al., 2017). The pharmacokinetic properties of the conjugated nanoparticle were tested in a mice experiment. The result shows that the conjugated nanoparticle had a prolonged circulation half-life (3 h) than unmodified drugs (about 10 min) (Mosafer et al., 2017). Besides, an increase in the cellular uptake of the conjugated nanoparticle in cancerous cells was also observed in cell experiments using flow cytometry. Thus, the conjugated nanoparticle exhibited an improvement in glioma cancer therapy.

PEGylation has long been widely used to extend the circulation half-life of drugs. However, since PEG is a synthetic polymer, many commercially available reagents are mixtures with different molecular weights, which leads to the non-uniform properties of pegylated drug products. In addition, the safety of PEG is still controversial. It is reported that taking PEGylated drugs may cause hypersensitivity reactions (Caballero et al., 2021), and the formation of antibodies against PEG results in the inefficacy of PEG to extend the biological half-life (Zhang et al., 2016). Moreover, PEG is difficult to metabolically excrete *in vivo*, causing bioaccumulation (Qian et al., 2019). These issues have prompted research and development of PEG alternatives to prolong biopharmaceuticals’ half-life. In addition to the aforementioned alternatives to PEG, attaching the nucleic acid aptamer to bioactive natural proteins is also a promising strategy.

#### 2.1.3 Albumin

Human serum albumin (HSA) is a protein abundantly present in human plasma (about 40 mg/ml) (Kitamura et al., 2014), which has a high binding affinity to various drugs. It has a molecular weight of 67 kDa and an attractive biological half-life of about 19 days (Hijazi, 2021). Albumin is the chief circulating protein which plays more and more important roles as a drug carrier in pharmaceutical uses nowadays (Larsen et al., 2016). The long blood circulation half-life of HSA, which facilitated by the engagement with the cellular recycling neonatal Fc receptor (FcRn), is a promising aptamer half-life extension strategy (Schelde et al., 2019). One basic aptamer delivery technology is to chemically coupling albumin with aptamers to form albumin-aptamer conjugates. Kuhlmann et al. (2017) developed a novel albumin-oligodeoxynucleotide assembly technology which provided a potential half-life extension strategy. This team utilized the 3′ or 5′ end maleimide-derivatized oligodeoxynucleotides to conjugate the albumin cysteine at position 34. This assembled construct showed stability in 10% serum over 24 h. Schmokel et al. (2017) also developed a strategy to conjugate an anticoagulant aptamer to recombinant albumins. This conjugation strategy was site-specific which was at a site distant from the binding pocket of HSA to FcRn. Thus, this technology retained the aptamer activity and albumin receptor interaction. Additionally, the binding affinity of the conjugate to FcRn was increased when the aptamer was conjugated to the recombinant albumin engineered for higher FcRn affinity. The strategies mentioned above provided possibilities to change the pharmacokinetic profile of aptamers.

#### 2.1.4 Alternatives to albumin

Besides albumin, other long-half-life proteins in human serum could be used to modify aptamers improving their pharmacokinetics properties. The constant fragment (Fc) domain of a human immunoglobulin (Ig) G also is used to improve the pharmacokinetics properties of biologically active proteins or peptides (Schellenberger et al., 2009). For example, in clinical trials, factor IX-Fc (Alprolix) exhibited a half-life of 57–83 h, which was threefold longer than other formulations of Factor IX alone (∼8 h) (Santagostino et al., 2012; Powell et al., 2013). The IgG has a higher circulation half-life than albumin *in vivo* (Ghetie and Ward, 2002; Roopenian and Akilesh, 2007). Additionally, a drug conjugated with the Fc domain of IgG results in higher pharmacokinetic properties than that of albumin. Moreover, the molecular weight of the Fc domain is close to either albumin or PEG.

Another protein that has been used to improve the half-life of drugs is transferrin. Human transferrin has a half-life reported to be 7–10 days or 10–12 days (Kontermann, 2009; Kim et al., 2010). The aglycosylated form of human transferrin could reach a half-life of 14–17 days (Kim et al., 2010). In the early 2000s, the transferrin fusion technology was used to develop long-acting drugs. For example, BRX-0585, a transferrin-glucagon-like peptide-1 (GLP-1) fusion protein for the treatment of type 2 diabetes mellitus (T2DM), demonstrated significantly enhanced half-life. Matsubara et al. also fused a DPP-4-resistant analog of GLP-1 to non-glycosylated transferrin (Matsubara et al., 2011). This molecule dramatically prolonged the half-life of GLP-1 from a few minutes to 27 h in rabbit model (Matsubara et al., 2011).

However, research works about using the Fc domain or transferrin conjugated/fusion to aptamers for extending circulation half-life have not been reported yet, so it is not clear whether the Fc domain or transferrin modified aptamers possess a longer elimination half-life than that of albumin. Generally speaking, aptamer drugs could only be administered by injection. However, the macromolecule moiety accounts for a large proportion within the macromolecule-aptamer conjugate, making it hard to increase the subcutaneous dosage for the aptamer moiety at a fixed subcutaneous administration volume, which dramatically limits the therapeutic potential. Therefore, the limitation of the space for the dosage increases caused by the macromolecular modification approach should be addressed.

### 2.2 Nucleic acid aptamer modified by low molecular weight coupling agents

**FIGURE 3:**
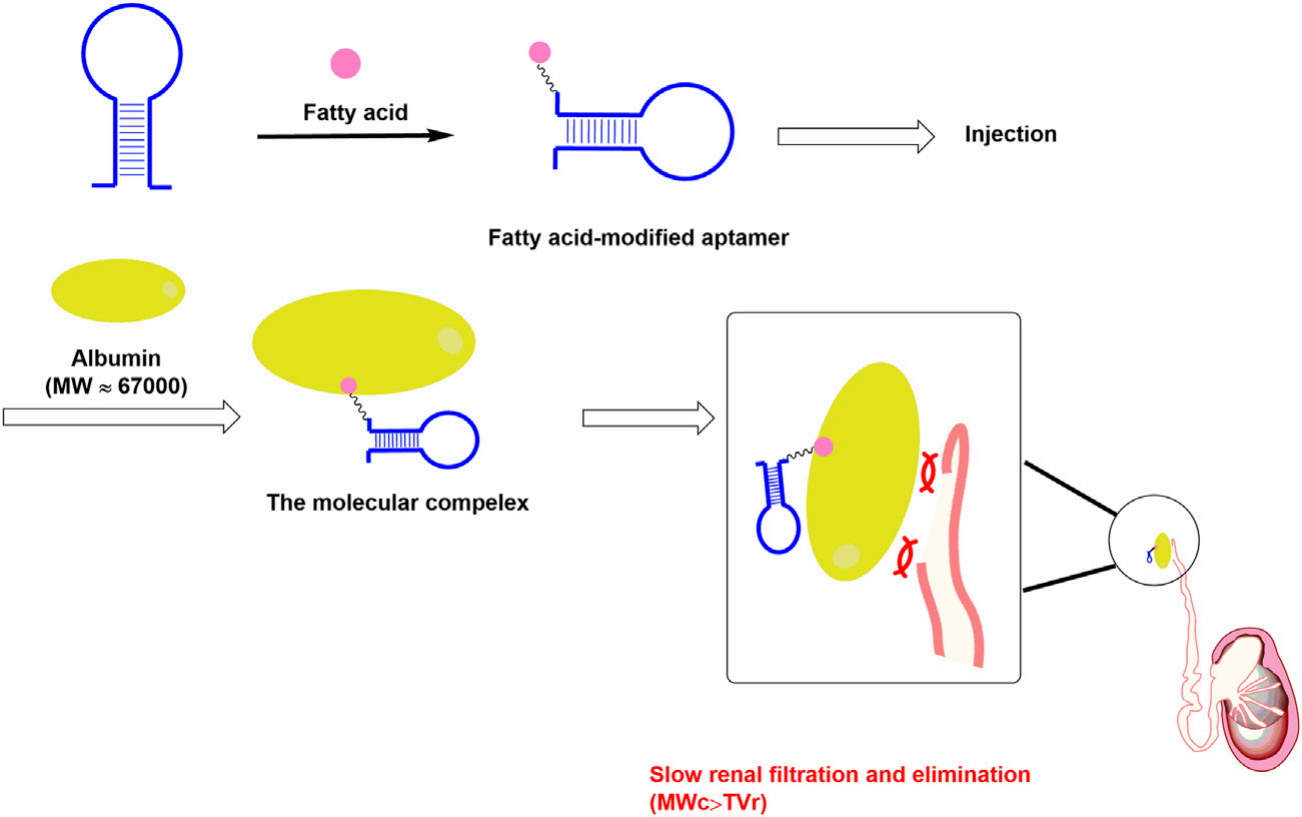
The design of low molecular modification approach for aptamer. Note: MW_c_ is short for the molecular weight of the above mentioned molecular complex (albumin@fatty acid-modified aptamer complex); TV_r_ is short for the cut-off threshold value of the renal filtration.

The second modification approach for aptamers is their conjugation to low-molecular-weight moieties, such as small molecules. As we mentioned above, albumin has large hydrophobic interface cages, which could bind some special LMWCAs to form molecular complexes. Mechanistically, some mentioned-above special LMWCAs could be used to modify aptamers in order to bind albumin to form molecular complexes with an average mass above the cut-off threshold of renal filtration for extending the circulation half-life (Liu et al., 2009) (Figure 3). This strategy could help drugs in attaching to albumin through non-covalent binding. It has been reported that Evans Blue (EB) could specifically bind to albumin with high binding affinity and has a prolonged circulation half-life *in vivo* (Liu et al., 2016). EB could insert into the hydrophobic moiety of albumin to form a drug/albumin complex. This complex exhibited longer circulation half-life and superior physiological stability. The EB derivatives have been widely used to modify either peptides or proteins to improve the pharmacokinetic profile of these drugs (Chen et al., 2016; Jacobson et al., 2016; Ehlerding et al., 2018). Recently, a team utilized this molecule to modify a PTK7 receptor-specific aptamer Sgc8 and got an EB-aptamer conjugate (Ding et al., 2020). This conjugate showed a significantly prolonged circulation half-life and improved targeting performance compared to the unconjugated one.

Another commonly used LMWCAs for extending circulation half-life are fatty acids (FAs). The majority of FAs could bind albumin with high affinity, and this property makes them proper agents for long-lasting modification. For example, the glucagon-like peptide-1 receptor agonists (GLP-1RAs), which were used for the treatment of type 2 diabetes, have been modified by several FAs to prolong the circulation half-life. The palmitic acid modified GLP-1RAs (liraglutide) possessed a circulation half-life of 3 days. Further, the next generation long-acting GLP-1RAs, semaglutide, which was modified by octadecandioic acid derivate, has an even longer circulation half-life of 7 days (Renaldi et al., 2021) and a decreased dosing frequency (Yu et al., 2022a). Due to the success of FA modification commercially, researchers have been trying to apply this strategy in the area of nucleic acid drugs, including aptamers. Jin et al. (Jin et al., 2018) used a lipid with two fatty acid derivate tails for the modification of floxuridine homomeric oligonucleotide (LFU20). This strategy could help LFU20 to insert into the hydrophobic cavity of albumin to form an LFU20/albumin complex, which facilitates LFU20 accumulation in tumors by the enhanced permeability and retention effect, and internalization into the lysosomes of cancer cells.

Recently, our research group screened out a FA derivative, which was used to conjugate the sclerostin aptamer. According to the results of the pharmacokinetics study, the circulation half-life of the modified sclerostin aptamer was 8 days in healthy rats (Number of patent application: PCT/CN 2022/082,996). Besides, in healthy rats, the circulation half-life of the PEGylated sclerostin aptamer and the marketed therapeutic sclerostin antibody romosozumab were merely 3 days. Therefore, the circulation half-life of fatty acid-modified sclerostin aptamer in healthy rats is at least 3-fold longer than either the circulation half-life of the PEGylated sclerostin aptamer or the marketed therapeutic sclerostin antibody romosozumab (Florio et al., 2016; Yu et al., 2022b; Wang et al., 2022). This long-lasting modification approach could not only increase the proportion of aptamer moiety within the conjugate and increase the therapeutic effect at a fixed subcutaneous administration volume, but also increase the treatment compliance of the aptamer drugs. According to the results of the pharmacodynamics study, both the fatty acid-modified sclerostin aptamer and the PEGylated sclerostin aptamer could increase bone mass and improve bone microarchitecture integrity in ovariectomy-induced osteoporotic rats at the same dosage and dosing interval. Importantly, the fatty acid-modified sclerostin aptamer demonstrated significantly better bone anabolic potential than the PEGylated sclerostin aptamer. This indicates that the prolonged circulation half-life and the high proportion of aptamer within the conjugate contribute to the therapeutic activity of the modified aptamer.

**FIGURE 4:**
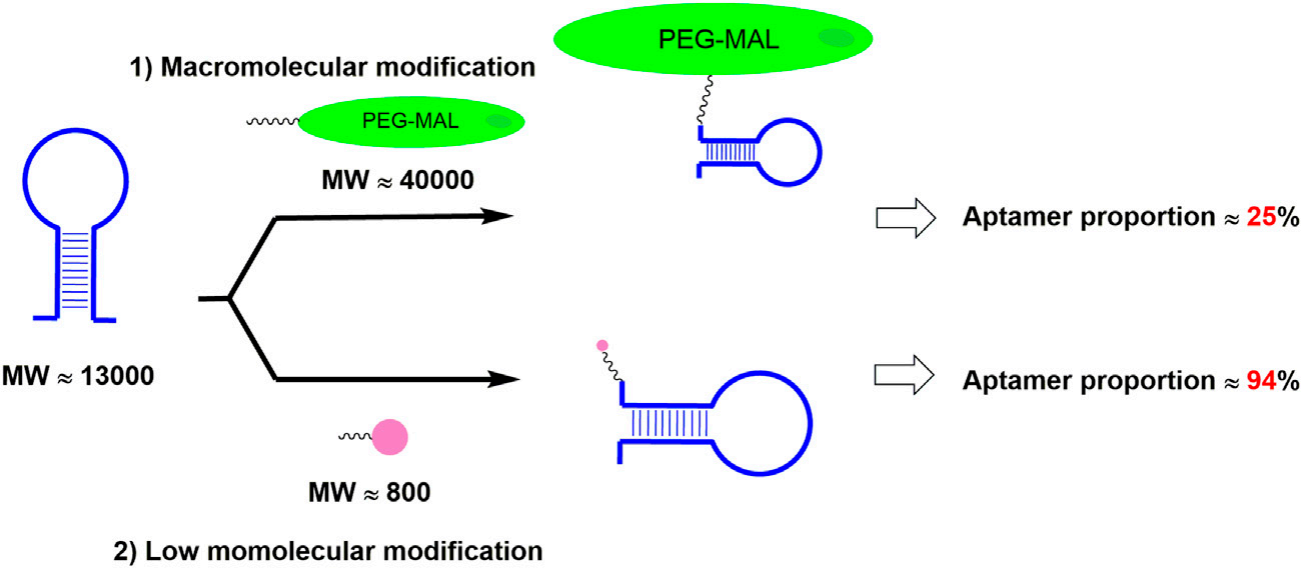
Different modification approach resulted in different aptamer proportion in the drug. Note: MW is short for the molecular weight; PEG-MAL is short for the polyethylene glycol maleimide.

Compared with PEGylation, the above-mentioned fatty acid modification approaches could greatly increase aptamer proportion (Figure 4). However, fatty acids could also bind to fatty acid-binding proteins (FABPs), which are widely distributed in various tissues of the body, resulting in the loss of the drugs in those tissues and then the decrease of the drug amounts in blood circulation (Hertzel and Bernlohr, 2000). In order to address the above issue, it is desirable to screen fatty acid derivatives with low binding affinity to FABP but high binding affinity to albumin.

## 3 Discussion and perspectives

### 3.1 The potential compliance concern caused by PEGylated aptamer therapy

Patients have preferred routes of therapeutic administration. Some people like oral drug therapy, while others accept injection therapy (Yu et al., 2022a). Injection therapy may cause compliance concerns in patients who prefer oral drug therapy to treat chronic diseases (Pons-Faudoa et al., 2019). Patients do not follow the medical treatment mainly because of frequent, uncomfortable, painful, and inconvenient treatment. For example, the compliance problem among patients with type 2 diabetes has been of great concern for a long time (Renaldi et al., 2021). The leading cause of this compliance concern is the frequent, uncomfortable, painful, and inconvenient insulin injections. Generally, the PEG component for clinical use can only extend the biological half-life of the therapeutic aptamer to no more than 3 days in most cases. Repeated subcutaneous injections at a short interval could largely reduce the clinical treatment compliance of the candidate aptamer drugs. To address this issue of treatment compliance, therapeutic aptamers could be modified by a long circulation half-life natural protein to possess a longer circulation half-life. They could be administrated by a new type of route that is comfortable, painless, and convenient.

### 3.2 The limited space for the dosage increases in aptamer modified by macromolecule

As we mentioned above, though PEGylation is a current modification approach to extend the circulation half-life of aptamers, the high molecular weight of PEG moiety occupies a large proportion within the PEG-aptamer conjugate, making the active aptamer moiety account for a very small proportion. Within a fixed subcutaneous administration volume in the clinical treatment, there is a serious limitation in subcutaneous dosage increase for the therapeutic aptamer, which dramatically limits the therapeutic potential. To increase the limited space for the dosage increases in PEGylated aptamer therapy, therapeutic aptamers with a higher proportion of active aptamer moiety need to be developed. As we mentioned above, the low molecular modification approach could not only increase the proportion of aptamer moiety within the conjugate but also increase the therapeutic effect at a fixed subcutaneous administration volume. Therefore, employing LMWCAs as the next generation of long-lasting modification approaches for aptamers is desirable. Also, therapeutic aptamers could be administrated by a new type of route that contains a larger dosage than the traditional subcutaneous route of administration.

### 3.3 Improving the administration method to increase dosage for aptamer drugs modified by macromolecule

Generally speaking, the volume of subcutaneous injection is less than 2 ml. For the aptamer drugs modified by macromolecule, the proportion of aptamer moiety is very low in the conjugate. Thus, an aptamer modified by a macromolecule could not achieve the same therapeutic effect compared to that modified by a low molecule at a fixed subcutaneous administration volume. To improve patient compliance and increase dosage limits of the aptamer drugs modified by macromolecule, the conventional hypodermic-needle injection method needs to be improved. A novel injector, dermojet, could inject high-velocity jets of the drug solution into the subcutaneous tissue (Jones et al., 2017; Barolet and Benohanian, 2018). This method is virtually painless, and the fluid of drug penetrate skin and underlying tissues at a very high velocity. Importantly, as this injector is needle-free, this technique is suitable for mass volume injections, which greatly increases the dose limit.

Another method to improve the injection volume is intravenous injection. The volume of intravenous injection could reach 10 ml, and this makes it possible for mass injection. Clinically, small doses of macromolecular drugs could be administered subcutaneously. However, high doses injection is usually done intravenously (Mccolm et al., 2014).

### 3.4 Developing AI-based strategies for optimization of low molecular weight coupling agents with high binding affinity to albumin

In addition to bind albumin, fatty acid could also bind to FABP, which are widely distributed in various body tissues, resulting in the loss of the drugs in those tissues and then the decrease of the drug amounts in blood circulation. Therefore, it is desirable to seek a FA derivative that could demonstrate low binding affinity to FABP but high binding affinity to albumin.

Warfarin was also reported to have a strong binding affinity to albumin, which can be used as the LMWCAs candidate. However, the inherent anticoagulation of warfarin raises the safety concerns of bleeding in some sensitive individuals, making warfarin unsuitable for application as a long-acting coupling agent. Therefore, seeking a coumarin analogue from warfarin derivatives with a high binding affinity to albumin and without an anticoagulant effect is desirable.

It was reported that the presence of fatty acids could facilitate the binding of warfarin to albumin. Therefore, it is desirable to develop a dual modification approach containing fatty acid derivative and warfarin derivative for aptamers. However, either octadecandioic acid or warfarin has lots of derivatives, all of which cannot be experimentally implemented due to the high labor and time cost. AI has witnessed successes in predicting the interactions between targets and ligands before final synthesis and verification in drug design (Paul et al., 2021). Thus, these molecules could be optimized through AI-based strategies to generate their derivatives with higher binding affinity to albumin and then predict the most promising dual combinations of both warfarin derivatives and fatty acid derivatives. Additionally, the synergistic effect could be enhanced by adding a suitable number of fatty acids to the binding pockets (Fujiwara and Amisaki, 2011). However, it is still unclear how many fatty acids could have a better effect on enhancing the binding of warfarin derivative to albumin. Therefore, optimizing the combinations of warfarin derivatives with different numbers of FAs for the pharmacokinetics-related aptamer optimization through AI-based strategies also is worth studying.
